# Forage grasses with lower uptake of caesium and strontium could provide ‘safer’ crops for radiologically contaminated areas

**DOI:** 10.1371/journal.pone.0176040

**Published:** 2017-05-01

**Authors:** Beth Penrose, Nicholas A. Beresford, Neil M. J. Crout, J. Alan Lovatt, Russell Thomson, Martin R. Broadley

**Affiliations:** 1 School of Biosciences, University of Nottingham, Sutton Bonington, Leicestershire, United Kingdom; 2 NERC Centre for Ecology & Hydrology, Lancaster, Lancashire, United Kingdom; 3 Institute of Biological, Environmental and Rural Sciences (IBERS), Aberystwyth University, Aberystwyth, Ceredigion, United Kingdom; 4 Science and Advice for Scottish Agriculture (SASA), Edinburgh, United Kingdom; United States Department of Agriculture, Agricultural Research Service, UNITED STATES

## Abstract

Substitution of a species or cultivar with higher uptake of an element by one with lower uptake has been proposed as a remediation strategy following accidental releases of radioactivity. However, despite the importance of pasture systems for radiological dose, species/cultivar substitution has not been thoroughly investigated for forage grasses. 397 cultivars from four forage grass species; hybrid ryegrass (*Lolium perenne* L. *x Lolium multiflorum* Lam.), perennial ryegrass (*Lolium perenne* L.), Italian ryegrass (*Lolium multiflorum* Lam.) and tall fescue (*Festuca arundinacea* Shreb.); were sampled from 19 field-based breeding experiments in Aberystwyth and Edinburgh (UK) in spring 2013 and analysed for caesium (Cs) and strontium (Sr) concentrations. In order to calculate concentration ratios (CRs; the concentration of an element in a plant in relation to the concentration in the soil), soils from the experiments were also analysed to calculate extractable concentrations of Cs and Sr. To test if cultivars have consistently low Cs and Sr concentration ratios, 17 hybrid ryegrass cultivars were sampled from both sites again in summer 2013 and spring and summer 2014. Tall fescue cultivars had lower Cs and Sr CRs than the other species. Three of the selected 17 hybrid ryegrass cultivars had consistently low Cs CRs, two had consistently low Sr CRs and one had consistently low Cs and Sr CRs. Cultivar substitution could reduce Cs CRs by up to 14-fold and Sr CRs by 4-fold in hybrid ryegrass. The identification of species and cultivars with consistently low CRs suggests that species or cultivar substitution could be an effective remediation strategy for contaminated areas.

## Introduction

Radiocaesium (^134^Cs and ^137^Cs) and radiostrontium (primarily ^90^Sr) are anthropogenic radionuclides that have been distributed in the environment due to nuclear weapons testing, nuclear power production and nuclear accidents [1]. Radioisotopes of Cs were the primary long-term contributors to human doses received after the Windscale [2](1957) and Chernobyl [3] (1986) accidents, and are now the principal radiological concern in areas affected by the accident at Fukushima in 2011 [4]. Strontium-90 is a major contributor to doses in areas contaminated by releases from the Mayak facility in the Russian Urals [5] and is present in soils of the Chernobyl Exclusion Zone at activity concentrations close to those of ^137^Cs [6]. These radionuclides are of particular concern as they are readily incorporated into biological systems due to their chemical similarity to the biologically essential elements potassium (K; radiocaesium) and calcium (Ca; radiostrontium). In the long term, radiocaesium and radiostrontium enter the human food chain mainly via consumption of crop plants grown on contaminated soils or products from animals fed on contaminated fodder.

A number of remediation strategies that aim to reduce the transfer of radionuclides to crop/fodder plants are available, and include mechanical soil amelioration techniques, chemical amendments and plant based strategies. Mechanical techniques such as ploughing can reduce transfer of Cs and Sr by 2–10 fold and chemical amendments such as the addition of organic matter, liming or application of fertiliser can reduce transfer by 1.3–3.0 fold, 1.5–4.0 fold and 1.5–3.0 fold, respectively [7]. For radiocaesium there are also highly efficient binders which can be orally administered to livestock to reduce absorption in the gastrointestinal tract and hence transfer to milk or meat [8]; however, this is not the case for radiostrontium[9]. Alternatively, plant based strategies are available; Cs and Sr uptake from soil can vary by two orders of magnitude between crop plant species [7] thus species substitution -where a crop species with high uptake of an element is replaced with a crop species with low uptake of an element- has the potential to cause a significant reduction in soil-plant transfer of these radionuclides [10]. However, changing production from one crop to another can be challenging; agronomic knowledge and a viable market must be available for the new crop [11][7]. Conversely, cultivar substitution for a given crop is likely to be readily applicable, socially acceptable and require no new skills, knowledge or markets. However, not enough is known about its efficacy for it to be widely recommended [12][13].

Ohlenshlaeger and Gissel-Nielson [14] [15] investigated variation in Cs uptake between two perennial ryegrass cultivars and found little variation (maximum 1.3) in radiocaesium soil-plant concentration ratios. It is likely that more variation would have been found if more cultivars were included in the study. Studies investigating inter-cultivar variation in uptake of chemical analogues of Cs and Sr (K, Ca and Mg) have shown that heritability of shoot concentrations of these elements is high in ryegrasses [16][17][18]; and fescues [16][17][19], and therefore breeding and selection of cultivars for low concentrations of these elements (and thus the analogous elements Cs and Sr) may be feasible [20].

Cultivars of Italian ryegrass [21] and tall fescue [22] with consistently high shoot Mg concentrations have been identified. However, other studies have found significant genotype x environment interactions for both ryegrasses [17] and fescues [17] [19] [18], and therefore cultivars with apparently high or low elemental shoot concentrations may behave inconsistently over multiple sites and/or years. Environmental factors can have a large effect on the uptake of Cs and Sr, and identifying cultivars that display consistently low concentration ratios over time and between sites has previously been challenging [12].

As forage grasses exhibit limited self-pollination, individuals of the same cultivar are not genetically identical, and are usually referred to as ‘populations’ by grass breeders. Although ‘cultivar’ is usually used to describe commercial varieties, the term ‘cultivar’ is used in this paper to describe non-commercial varieties in addition to commercial varieties.

The aim of the study described was to investigate the variation in stable caesium (Cs) and strontium (Sr) uptake between 397 cultivars from four commonly used forage grass species using measurements from a programme of long term grass breeding experiments. Particular attention was paid to the identification of cultivars that have consistently low Cs and/or Sr concentration ratios at both sites, over two study years and from both spring and summer harvests.

## Materials and methods

### Overview

Caesium and strontium analyses were made on samples of 397 cultivars from four forage grass species. The grass species were: hybrid ryegrass (*Lolium perenne* L. x *Lolium multiflorum* Lam.), perennial ryegrass (*Lolium perenne* L.), Italian ryegrass (*Lolium multiflorum* Lam.) and tall fescue (*Festuca arundinacea* Shreb.). Samples were collected from established breeding experiments in Aberystwyth, UK and Edinburgh, UK, in the spring and summer of 2013 and 2014. Due to the ongoing structure of the ongoing breeding experiments not all cultivars were available for sampling at each site or time period. As one of our key objectives was to test whether cultivars behaved similarly in terms of Cs and Sr uptake over different seasons and in multiple years, we organised our work to maximise the number of samples from each site and time period.

### Experimental design

The breeding programme is structured into individual experiments, each of which consisted of between 40 and 416 plots (Table 1). with between 10 and 104 cultivars grown in a randomised block design in a given field and sown between one and three years prior to our sampling.

**Table 1:** Sowing and harvest dates, number of cultivars, replicates, plots, cuts per year and the plot area in each of the 19 experiments sampled in this study.

Experiment number	Site	Field number	Sowing date	Plot area (m^2^)	No. of plots	No. of cultivars	No. of replicates of each cultivar sown	No. of cuts in 2013	No. of cuts in 2014	Spring cut 2013	Summer cut 2013	Spring cut 2014	Summer cut 2014
**1**	**Aberystwyth**	1	10/08/10	2	68	17	4	7	7	20/05/13	-	-	-
**2**	**Edinburgh**	4	16/08/10	2	68	17	4	7	7	30/05/13	-	-	-
**3**	**Aberystwyth**	1	10/08/10	1	64	16	4	7	7	20/05/13	-	-	-
**4**	**Aberystwyth**	1	16/08/10	1	40	10	4	7	7	03/06/13	-	-	-
**5**	**Aberystwyth**	1	10/08/10	1	72	18	4	7	7	20/05/13	-	-	-
**6**	**Edinburgh**	4	16/08/10	1	72	18	4	7	7	03/06/13	-	-	-
**7**	**Aberystwyth**	2	08/08/11	2	152	38	4	7	7	20/05/13	09/09/13	29/05/14	28/08/14
**8**	**Edinburgh**	4	22/08/11	2	100	25	4	7	7	28/05/13	27/08/13	19/05/14	29/08/14
**9**	**Aberystwyth**	2	08/08/11	4.68	108	27	4	9	5	03/06/13	-	-	-
**10**	**Aberystwyth**	2	08/08/11	4.68	76	19	4	9	5	04/06/13	-	-	-
**11**	**Aberystwyth**	2	08/08/11	4.68	40	10	4	6	6	03/06/13	-	-	-
**12**	**Aberystwyth**	3	14/09/12	1.2	84	21	4	7	7	04/06/13	-	-	-
**13**	**Aberystwyth**	3	14/09/12	2	248	62	4	7	7	22/05/13	29/08/13	15/05/14	12/08/14
**14**	**Aberystwyth**	3	13/09/12	4.68	108	27	4	5	9	22/05/13	-	-	-
**15**	**Edinburgh**	4	20/08/12	2	64	16	4	7	7	22/05/13	23/08/13	19/05/14	25/08/14
**16**	**Aberystwyth**	3	13/09/12	4.68	80	20	4	5	9	30/05/13	-	-	-
**17**	**Aberystwyth**	3	14/09/12	1.2	416	104	4	7	7	31/05/13	-	-	-
**18**	**Edinburgh**	4	20/08/12	2	126	42	3	7	7	12/06/13	-	-	-
**19**	**Edinburgh**	4	21/08/12	2	126	42	3	7	7	13/06/13	-	-	-

The Aberystwyth experiments were located in three fields at the Gogerddan Campus of the Institute of Biological and Environmental Sciences (IBERS). Field 1 (52°25'40.1"N 4°01'25.5"W) contained 2010-sown plots, Field 2 (52°26'01.1"N 4°00'42.1"W) the 2011-sown plots and Field 3 (52°26'03.6"N 4°00'27.5"W) the 2012-sown plots. The Edinburgh experiments at the Roddinglaw site of Science and Advice for Scottish Agriculture (SASA) (55°55’40.9”N, 3°20’25.3”W),. The distribution of cultivars and species between the sampled experiments is given in Table 2.

**Table 2:** Number of different cultivars grown in Aberystwyth, Edinburgh and in total.

Site	Hybrid ryegrass	Perennial ryegrass	Italian ryegrass	Tall fescue
**Aberystwyth**	100	189	8	10
**Edinburgh**	29	100	16	0
**Total number**	101 (28)	269 (20)	17 (7)	10 (0)

Number in parenthesis are the number of cultivars shared between both site.

The cutting regime varied between experiments depending on the requirements of the breeding programme (Table 1). All cuts were carried out between April and November which is typical for UK grassland management (Table 1).

Not all of the samples from these replicates could be analysed, and our results are derived from between 1 and 4 replicates of each cultivar per experiment.

### Preparation of and maintenance of experiment plots

The plots were ploughed using a 5-furrow reversible plough prior to sowing. Experiments 1, 3, 7, 9, 10, 11, 13, 14 and 16 were drill sown. The rest of the experiments were broadcast sown. Fertilisers were applied before the first cut and after each cut (Table 3) in all experiments apart from 14 and 16, where the fertiliser was applied twice prior to the first cut. using a calibrated drop spreader (Gandy, Minnesota, USA).

**Table 3:** Fertiliser treatments for the 19 experiments.

Experiments	Fertiliser before cut 1 (kg ha^-1^)	Fertiliser after cut 1 (kg ha^-1^)	Fertiliser after cut 2 (kg ha^-1^)	Fertiliser after cut 3 (kg ha^-1^)	Fertiliser after cut 4 (kg ha^-1^)	Fertiliser after cut 5 (kg ha^-1^)	Fertiliser after cut 6 (kg ha^-1^)	Fertiliser after cut 7 (kg ha^-1^)	Fertiliser after cut 8 (kg ha^-1^)	Fertiliser after cut 9 (kg ha^-1^)
**4,5,12,17**	57	100	57	57	57	57	57	57 *	-	-
**1,3,7,13,**	57	100	100	57	57	57	57	57 *	-	-
**2,6,8,15,18,19**	53	105	79	53	53	53	53	-	-	-
**9, 10**	57	35	35	35	35	35	35	35	35	57*
**14, 16**	57 (twice)	86	86	35	35	57 *	-	-	-	-
**11**	57	57	57	35	57	57	57 *	-	-	-

All fertilisers are shown in kg ha^-1^. Sulphurcut (YaraMila, Grimsby UK) of 23:4:13 N:P_2_O_5_:K_2_O) + 7% SO_3_ was used except for when the applications are denoted with *, where the fertiliser was 0:24:24 (N:P_2_O_5_:K_2_O) + 7% SO_3_.

### Sample collection and preparation

The plots were cut to approximately 5 cm height using a custom Haldrup grass harvester (Haldrup GmbH, Ilshofen, Germany) as part of the normal operations of the breeding programme. For our work a subsample (200–320 g fresh mass) of sward was taken by hand from cuttings ejected from the harvester. Samples were collected and oven dried (80–90°C) for 2–5 days depending on the moisture content of the samples. Grass samples were then ground using a cutting mill, and *c*. 10 g dry mass (DM) of this ground sample was retained for further analyses.

A washing test was undertaken to determine whether the concentrations of Cs and Sr were affected by washing (see S1 Table and S1 Fig). On the basis of this test samples were not washed before drying thereby reducing the sample preparation workload.

Soil properties were measured using five soil cores of 10–15 cm depth from each experimental area, taken in a W-pattern using a soil auger between June 2013 and March 2014. The five cores were homogenised and bulked to form one sample per experiment. The pH_water_ was measured using a HI 9024 microcomputer pH meter (Hanna Instruments, Padova, Italy) using the method described by Emmett *et al*. [23]. To determine moisture content, soils were weighed (wet mass) and air-dried at ~30^°^C for 3–11 weeks then oven dried at 80°C for 3–4 days, after which they were reweighed (dry mass). Approximately 10 g was subsampled from the bulk, and ground in a ball mill using agate bowls and balls. Soil type was determined using the soil texture layer on the UKSO soils map viewer [24].

### Acid digestion of grass samples

A subsample of ~0.3 g DM of ground grass was weighed into a digestion tube and 2 mL of concentrated nitric acid (80% trace element grade HNO_3_), 1 mL of hydrogen peroxide (>30% H_2_O_2_) and 1 mL of ultra-pure ‘Milli-Q’ water (18.2 MΩ cm; Fisher Scientific UK Ltd, Loughborough, UK) added. These tubes were placed into a microwave digester (Multiwave 3000 with 48-vessel 48MF50 rotor; Anton Paar GmbH, Graz, Austria) with microwave settings as follows: power = 1400 W, temperature = 140°C, pressure = 2 MPa, time = 40 minutes. Once digested, each sample was diluted with 11 mL of ultra-pure water. An aliquot of 2 mL was then removed from this sample, and diluted with a further 8 mL of ultra-pure water prior to analysis using inductively-coupled plasma mass spectrometry (ICP-MS).

### Soil ammonium nitrate extraction

Ammonium nitrate (NH_4_NO_3_) is commonly used to extract minerals from soil, and this ‘extractable fraction’ is considered to be a measure of what is available to the plant [25]. To determine extractable mineral concentrations approximately 2 g of dry soil was taken from the milled samples from each experiment, and weighed into centrifuge tubes. 20 mL of 1 M NH_4_NO_3_ was added to each tube. Tubes were then agitated using an end-over-end shaker for ~12 h overnight. The tubes were then centrifuged at 2500 rpm for 10 minutes and 10 mL of the supernatant was passed through a 22 μm syringe-driven filter into a universal tube. 1 mL of the filtered supernatant was then transferred via pipette into ICP tubes and diluted with 9 mL of 2% nitric acid ready for analysis using ICP-MS.

### Mineral analysis

Multi-element analysis of diluted solutions was undertaken by ICP-MS. Samples from the spring cut 2013 of Experiments 1, 2, 4, 7, 11, 13, 14, 15, 16 and 18 were analysed using a Thermo-Fisher Scientific X-Series^II^ instrument (Thermo Fisher Scientific Inc., Waltham, MA, USA). All other samples from the spring cut 2013, summer cut 2013, spring cut 2014 and summer cut 2014 were analysed using a Thermo-Fisher Scientific iCAP-Q instrument (Thermo Fisher Scientific, Bremen, Germany), with appropriate cross-calibration between instruments. Detailed information about the ICP-MS methods used can be found in S1 Table.

### Data analysis

All data analyses were conducted using R [26]. We interpreted Cs and Sr uptake by calculating concentration ratio (CR; Eq 1) for each of the grass samples using the concentration of the element in the grass divided by the corresponding ammonium nitrate extractable soil concentration. The CR is an internationally accepted approach for radionuclide uptake assessment (e.g. [27]) here we are using it to account for variation in soil concentration of Cs and Sr in across our experimental locations. Neither Cs or Sr are essential elements and there is no evidence to suggest that their *transfer* from soil to grass is dependent upon soil Cs or Sr concentrations [28]; we acknowledge that CR varies depending upon soil type [2] [27] but this does not negate the use of CRs for the purposes of this assessment.

Where a cultivar was grown in multiple experiments at the same site, mean Cs and Sr CRs for that cultivar were calculated using concentration ratios from all the Experiments at that site. All arithmetic means are denoted by $\bar{x}$.

$$Concentration\;ratio=\frac{Concentration\;of\;element\;in\;the\;plant\;(mg\;{kg}^{-1}\;DW)}{Concentration\;of\;element\;in\;the\;soil\;(mg\;{kg}^{-1\;}DW)}$$ (1)

Inter-cultivar variation, the difference between the concentration of an element in the highest accumulating cultivar and the lowest accumulating cultivar shows the full extent of variation for a group of cultivars. In this study, inter-cultivar variation was calculated using Eq (2).

$$Inter-cultivar\;variation=\frac{Mean\;concentration\;in\;the\;highest\;accumulating\;cultivar}{Mean\;concentration\;in\;the\;lowest\;accumulating\;cultivar}$$ (2)

## Results

### Between species variation in mean Cs concentration ratios

**Fig 1:**
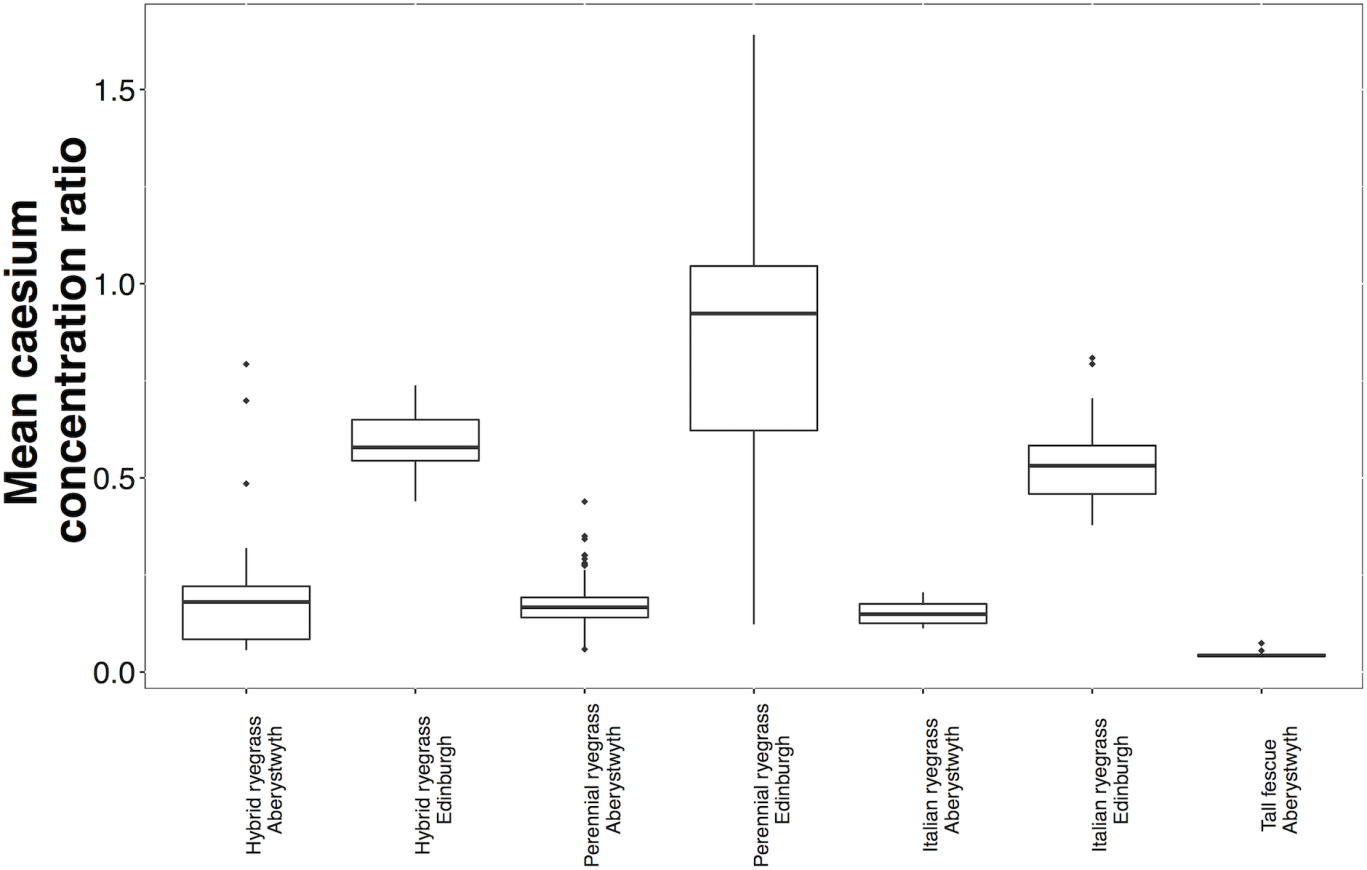
Mean caesium concentration ratios for hybrid ryegrass cultivars (Aberystwyth, number of cultivars = 100; Edinburgh, number of cultivars = 29), perennial ryegrass cultivars (Aberystwyth, number of cultivars = 189; Edinburgh, number of cultivars = 100), Italian ryegrass cultivars (Aberystwyth, number of cultivars = 8; Edinburgh, number of cultivars = 16) and tall fescue cultivars (Aberystwyth, number of cultivars = 10). The upper and lower ‘hinges’ refer to the 25^th^ and 75^th^ percentiles, the limits of the upper and lower whiskers refer to data that fall within 1.5* the inter-quartile range (IQR). The outliers (signified by ◆) show data outside this 1.5*IQR.

Tall fescue cultivars had a lower median Cs CR (0.0424) than median Cs CRs in hybrid ryegrasses, perennial ryegrass cultivars and Italian ryegrass cultivars grown in Aberystwyth, which were similar (0.180, 0.167, 0.149, respectively; Fig 1). Median Cs CRs were similar for hybrid ryegrass (0.578) and Italian ryegrass (0.531) and were higher in perennial ryegrass (0.923) cultivars grown in Edinburgh (Fig 1).

### Inter-cultivar variation in mean Cs concentration ratios

Caesium CRs varied by 14-fold (range = 0.06–0.79) between the 100 hybrid ryegrass cultivars grown in Aberystwyth (Fig 1), and approximately 2-fold (range = 0.44–0.74) between the 29 hybrid ryegrass cultivars grown in Edinburgh. Caesium CRs for perennial ryegrass grown in Aberystwyth (189 cultivars) and Edinburgh (100 cultivars) varied by 7-fold (range = 0.06–0.44) and 13-fold (range = 0.12–1.64), respectively (Fig 1).

Less variation was observed between the Italian ryegrass and tall fescue cultivars sampled (Fig 1); Cs CRs varied by only ≈2-fold in the eight Italian ryegrass cultivars grown in Aberystwyth (range = 0.11–0.21), the 16 Italian ryegrass cultivars grown in Edinburgh (range = 0.38–0.81) and the 10 tall fescue varieties grown in Aberystwyth (range = 0.37–0.74).

### Between species variation in mean Sr concentration ratios

Aberystwyth-grown hybrid ryegrass, perennial ryegrass and Italian ryegrass median Sr CRs were similar (1.64, 1.49 and 1.68, respectively) and were greater than the median Sr CR for tall fescue (1.00). Edinburgh-grown hybrid ryegrass had a lower median CR (0.71) when compared to Italian ryegrass (1.03), which had a lower Sr CR than the perennial ryegrass cultivars (1.20).

### Inter-cultivar variation in mean Sr concentration ratios

**Fig 2:**
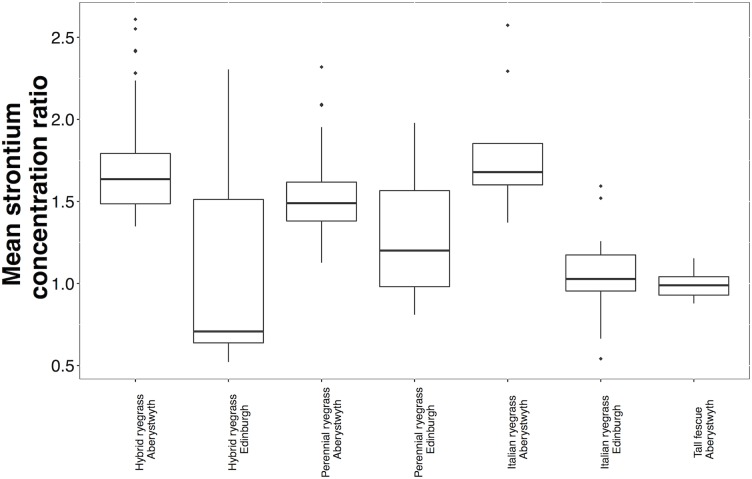
Mean strontium concentration ratios for hybrid ryegrass cultivars (Aberystwyth, number of cultivars = 100; Edinburgh, number of cultivars = 29), perennial ryegrass cultivars (Aberystwyth, number of cultivars = 189; Edinburgh, number of cultivars = 100), Italian ryegrass cultivars (Aberystwyth, number of cultivars = 8; Edinburgh, number of cultivars = 16) and tall fescue cultivars (Aberystwyth, number of cultivars = 10). The upper and lower ‘hinges’ refer to the 25^th^ and 75^th^ percentiles, the limits of the upper and lower whiskers refer to data that fall within 1.5* the inter-quartile range (IQR). The outliers (signified by ◆) show data outside this 1.5*IQR.

The 29 hybrid ryegrass cultivars, 100 perennial ryegrass cultivars and 16 Italian ryegrass cultivars grown in Edinburgh displayed more variation in strontium CRs (4.4-fold (range = 0.325–1.00); 2.5-fold (range = 0.482–2.28) and 2.9-fold (range = 0.285–1.10) respectively; Fig 2) than those grown in Aberystwyth (hybrid ryegrass: 1.9-fold, (range = 1.35–2.61; number of cultivars = 100); perennial ryegrass: 2.1-fold, (range = 1.13–2.32; number of cultivars = 189) and Italian ryegrass cultivars 1.9-fold, (range = 1.37–2.57; number of cultivars = 8), Fig 2). The 10 tall fescue cultivars grown in Aberystwyth varied less (1.3-fold; range = 0.88–1.15).

### Variance component analysis

Variance components were calculated using the mixed effects model *lme4* package [29] using data from spring cut 2013. All the Cs and Sr CRs were natural log transformed, so the residuals were distributed normally. Concentration ratio (Cs/Sr), cultivar, species and experiment location were classed as random variables in the model. Cultivar was nested within species, and experiment number was nested within experiment location in the model. Environmental factors such as experiment location and experiment number explain a large amount of the variation in both Cs and Sr CRs (Table 4). Variety explains more of the variation in Cs (~3%) than Sr (~0.8%) CRs, whereas the opposite is true for species (Cs = >0.001%; Sr = ~0.4%).

**Table 4:** Variance component analysis of caesium and strontium concentration ratios from spring cut 2013.

**Caesium**	** **	** **	** **
	**Variance**	**Std Dev**	**% Variation**
Experiment number	0.0768	0.2771	57.2
Experiment location	0.0367	0.00151	27.3
Variety	0.00417	0.06456	3.1
Species	1.12 x 10^−15^	3.35 x 10^−8^	8.37 x 10^−13^
Residuals	0.0166	0.129	12.4
**Strontium**			
Experiment number	0.287	0.536	23.5
Experiment location	0.833	0.912	68.0
Variety	0.00949	0.0974	0.8
Species	0.00431	0.0656	0.4
Residuals	0.0909	0.302	7.4

### Site characteristics

The soil from Aberystwyth was characterised as sand to sandy loam, and the soil from Edinburgh as clay loam to sandy loam. The soil at both sites was classified as moderately acidic [30], as the pH ranged from 4.8–6.6 ($\bar{x}$ = 5.8) in Aberystwyth and from 5.3–6.4 ($\bar{x}$ = 5.9) in Edinburgh (Table 5). Soil moisture contents were similar for both sites, ranging from 13.8–18.3% ($\bar{x}$ = 16.5%) in Aberystwyth and 16.8–19.3% ($\bar{x}$ = 18.2%) in Edinburgh.

**Table 5:** Sampling data, pH, moisture content (%), extractable Cs, Sr, K and Ca concentrations (mg kg^-1^) in the soils from the 19 experiments in Aberystwyth and Edinburgh.

Experiment number	Site	Sampling date	pH	Moisture (%)	Extractable Cs concentration (mg kg^-1^)		Extractable Sr concentration (mg kg^-1^)	Extractable K concentration (mg kg^-1^)	Extractable Ca concentration (mg kg^-1^)
**1**	Aberystwyth	03/07/2013	4.8	15.6		0.0699	3.94	118	1010
**2**	Edinburgh	01/11/2013	5.3	18.0		0.0116	14.4	122	1520
**3**	Aberystwyth	03/07/2013	5.0	13.8		0.0849	4.92	154	1610
**4**	Aberystwyth	04/03/2014	5.1	18.3		0.0276	12.6	128	1540
**5**	Aberystwyth	03/07/2013	5.5	17.9		0.0862	4.77	90.7	1230
**6**	Edinburgh	0/11/2013	5.9	18.0		0.0116	14.4	122	1520
**7**	Aberystwyth	03/07/2013	6.3	17.2		0.0909	3.47	177	1620
**8**	Edinburgh	13/06/2014	6.4	19.3		0.0082	25.5	98.5	1670
**9**	Aberystwyth	03/07/2013	5.1	16.3		0.0872	3.87	168	1810
**10**	Aberystwyth	03/07/2013	5.7	18.2		0.0875	4.15	143	1810
**11**	Aberystwyth	03/07/2013	5.7	17.7		0.0935	4.16	191	1910
**12**	Aberystwyth	03/07/2013	6.1	16.5		0.0954	4.71	109	1740
**13**	Aberystwyth	03/07/2013	6.9	15.3		0.0965	5.23	107	1810
**14**	Aberystwyth	03/07/2013	6.6	15.7		0.0825	5.75	123	2070
**15**	Edinburgh	01/11/2013	5.5	16.8		0.0116	13.9	140	1530
**16**	Aberystwyth	03/07/2013	6.3	16.4		0.0900	5.54	127	1880
**17**	Aberystwyth	03/07/2013	6.2	16.0		0.103	5.53	128	1870
**18**	Edinburgh	01/11/2013	6.2	18.0		0.0128	14.6	86.2	1800
**19**	Edinburgh	01/11/2013	5.8	19.0		0.0102	11.2	128	1370

Soil extractable Cs was higher in Aberystwyth (range = 0.0166–0.103; $\bar{x}$ = 0.084 mg kg^-1^) than in Edinburgh (range = 0.00824–0.0128; $\bar{x}$ = 0.011 mg kg^-1^), though the converse was true for extractable Sr; the extractable Sr concentration in Aberystwyth (range = 3.47–12.6; $\bar{x}$ = 5.28 mg kg^-1^) was almost three times lower than that in Edinburgh, (range = 11.2–25.5; $\bar{x}$ = 15.7 mg kg^-1^; Table 5). Concentrations of extractable Ca and K were relatively similar between the two sites (*K*: Aberystwyth, range = 90.7–191 mg kg^-1^, $\bar{x}$ = 136 mg kg^-1^; Edinburgh, range = 86.2–139 mg kg^-1^, $\bar{x}$ = 116 mg kg^-^; *Ca*: Aberystwyth, range = 1010–2070 mg kg^-1^, $\bar{x}$ = 1680 mg kg^-1^; Edinburgh, range = 1370–1790 mg kg^-1^ $\bar{x}$ = 1570; Table 5).

### Consistency of inter-cultivar variation between cuts and locations

**Fig 3:**
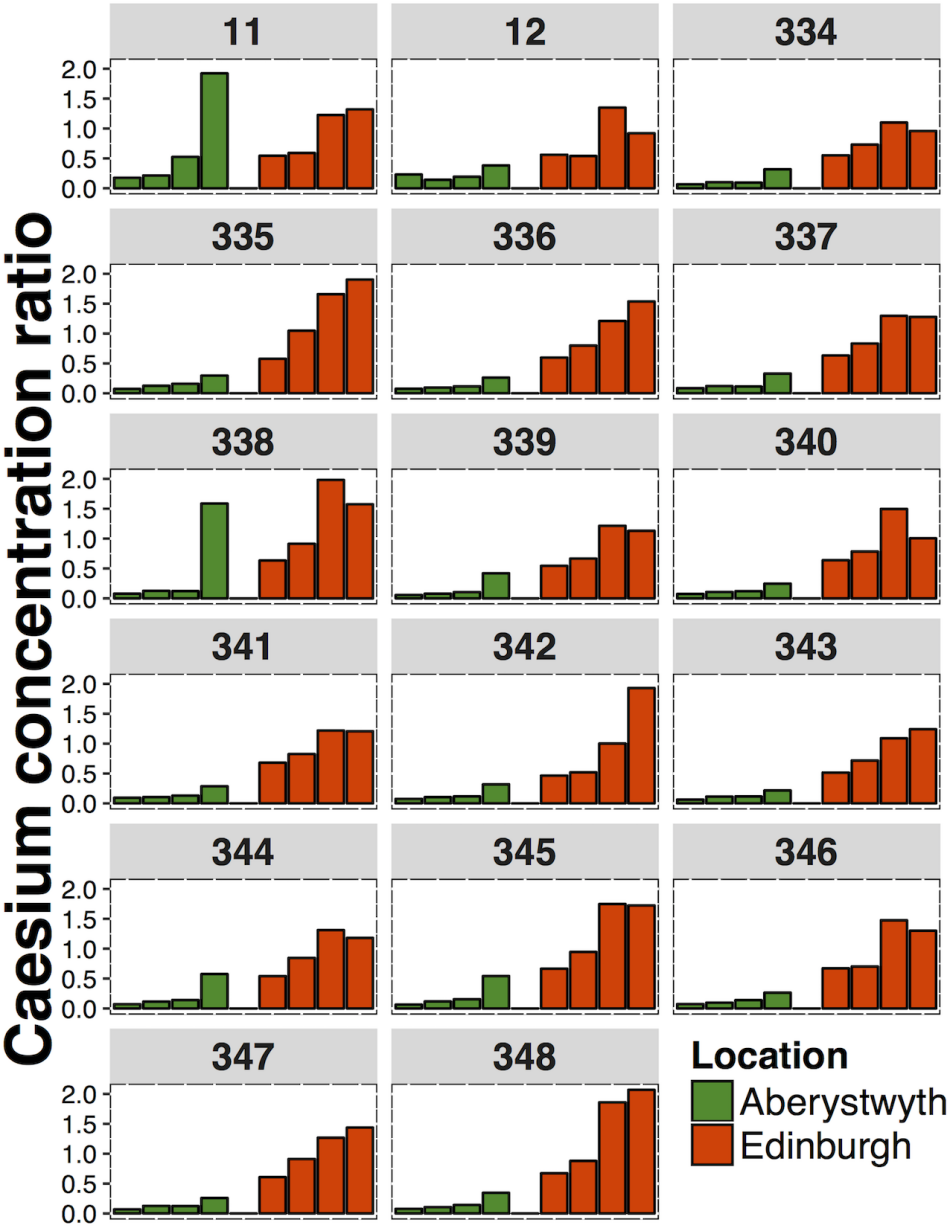
Mean caesium concentration ratios of 17 hybrid ryegrass cultivars from Aberystwyth and Edinburgh. Individual bars from left-right represent the: spring cut 2013, summer cut 2013, spring cut 2014, summer cut 2014. The numbers above each panel refers to the cultivar number.

To compare the consistency of inter-cultivar variation in Cs and Sr uptake over time and between sites, the 17 hybrid ryegrass cultivars that were grown in both Aberystwyth and Edinburgh were selected from the larger population shown in Figs 1 and 2. These cultivars (Table 6) were sampled in spring and summer (spring = May-June, summer = August-September; Table 1) in both 2013 and 2014. The Cs and Sr CRs were estimated using the same method as above. Caesium and strontium CRs were higher in the summer cuts of these 17 hybrid ryegrass cultivars than in the spring cuts in every location and 2014 harvests tended to have higher Cs and Sr concentration ratios than 2013 harvests (Fig 3A and 3B).

**Table 6:** Experiment numbers of the 17 hybrid ryegrass cultivars grown in Aberystwyth and Edinburgh.

Cultivar number	Experiment numbers Aberystwyth	Experiment numbers Edinburgh
**11**	1, 3, 7, 13	8, 15, 2
**12**	7, 13	8, 15
**334**	7, 13	8, 15
**335**	7, 13	8, 15
**336**	7, 13	8, 15
**337**	7, 13	8, 15
**338**	7, 13	8, 15
**339**	7, 13	8, 15
**340**	7, 13	8, 15
**341**	7, 13	8, 15
**342**	7, 13	8, 15
**343**	7, 13	8, 15
**344**	7, 13	8, 15
**345**	7, 13	8, 15
**346**	7, 13	8, 15
**347**	7, 13	8, 15
**348**	7, 13	8, 15

**Fig 4:**
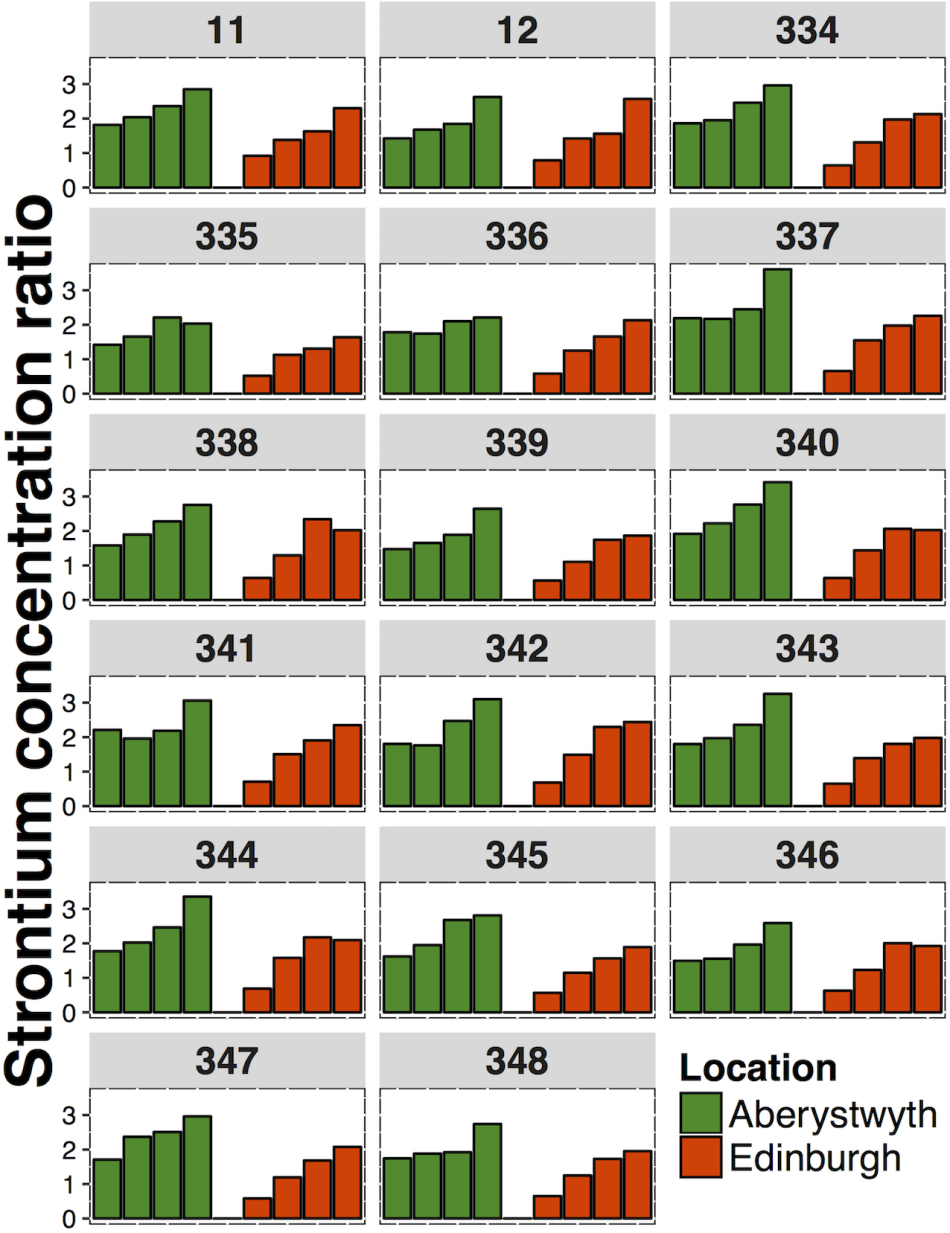
Mean strontium concentration ratios of 17 hybrid ryegrass cultivars from Aberystwyth and Edinburgh. Individual bars from left-right represent the: spring cut 2013, summer cut 2013, spring cut 2014, summer cut 2014. The numbers above each panel refers to the cultivar number.

The inter-varietal variation in Cs CRs for the 17 selected hybrid ryegrass cultivars ranged between 1.46 (spring cut 2013, Edinburgh) and 8.85 (summer cut 2014, Aberystwyth). For all cuts, the inter-varietal variation in Cs CRs was higher in Aberystwyth than in the equivalent cut in Edinburgh (Fig 3). The inter-varietal variation in Sr CRs (Fig 4) ranged from 1.43 (spring cut 2014, Edinburgh) to 1.79 (summer cut 2013, Edinburgh).

### Consistency of lower concentration ratios between cuts and locations

The mean Cs CR and mean Sr CR of the 17 hybrid ryegrass cultivars in each of the eight site/harvest combinations were ranked. These ranks were then summed to create a total rank for each cultivar (S3 Table).

The total ranks were compared to a distribution of random ranks. This distribution was created by summing eight (one for each location-harvest combination) randomly-generated numbers between one and 17 to create a simulated ‘total rank’. This process was iterated 1000 times, and the 5^th^ and 95^th^ percentile were calculated. Any cultivar with a total rank below the 5^th^ percentile was considered significantly consistently low-ranking, and any cultivar with a total rank above the 95^th^ percentile was deemed significantly consistently high-ranking.

Two cultivars (335 and 346), were identified as having consistently lower Sr CRs and three cultivars (334, 342 and 343) were identified as having consistently lower Cs CRs. One cultivar (339) had consistently lower Cs and Sr CRs (S3 Table in the Supplementary Information). Five cultivars were identified as having consistently higher Sr CRs (337, 340, 341, 342 and 344) and four cultivars were identified as having consistently higher Cs CRs (335, 338, 345 and 348).

### Influence of study size on species-wide variation in Cs and Sr concentration ratios

**Fig 5:**
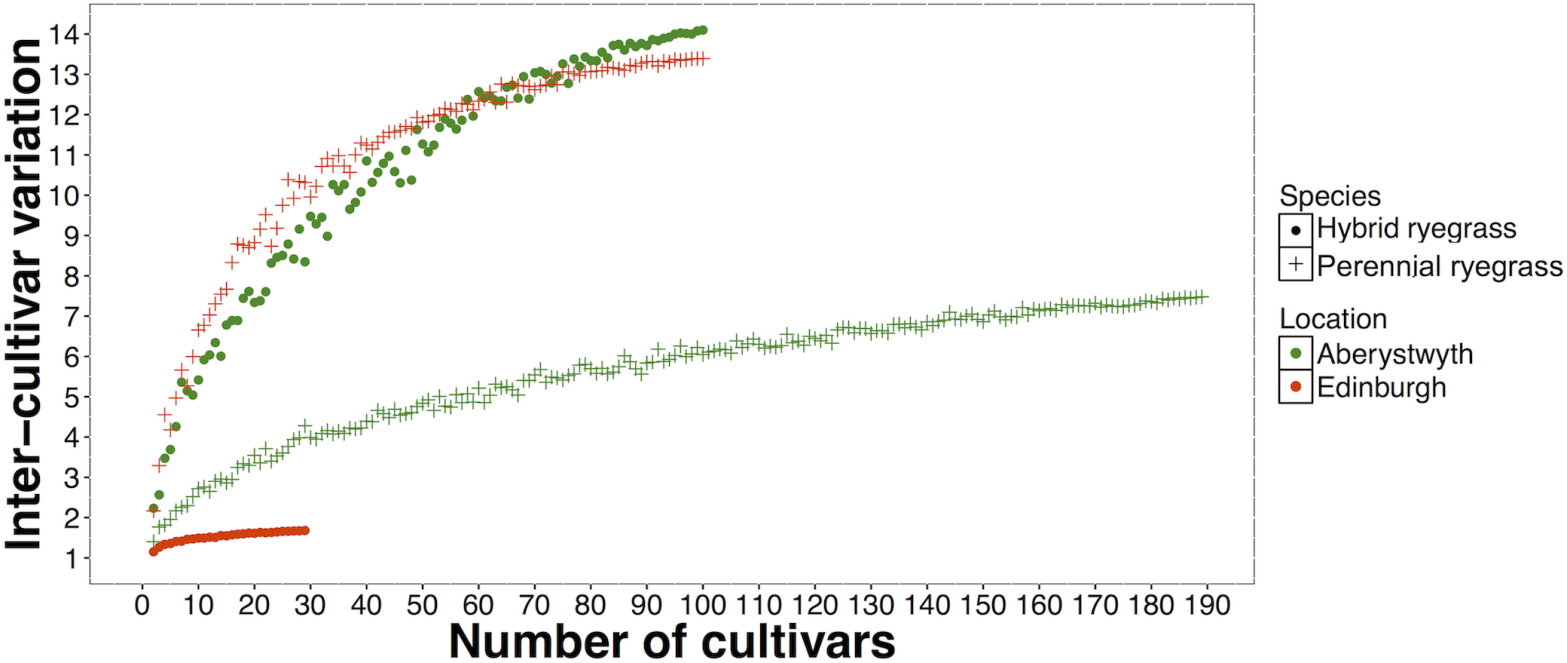
Inter-cultivar variation in caesium concentration ratios for 2 to maximum number of cultivars. Mean value of 100 simulations is shown.

**Fig 6:**
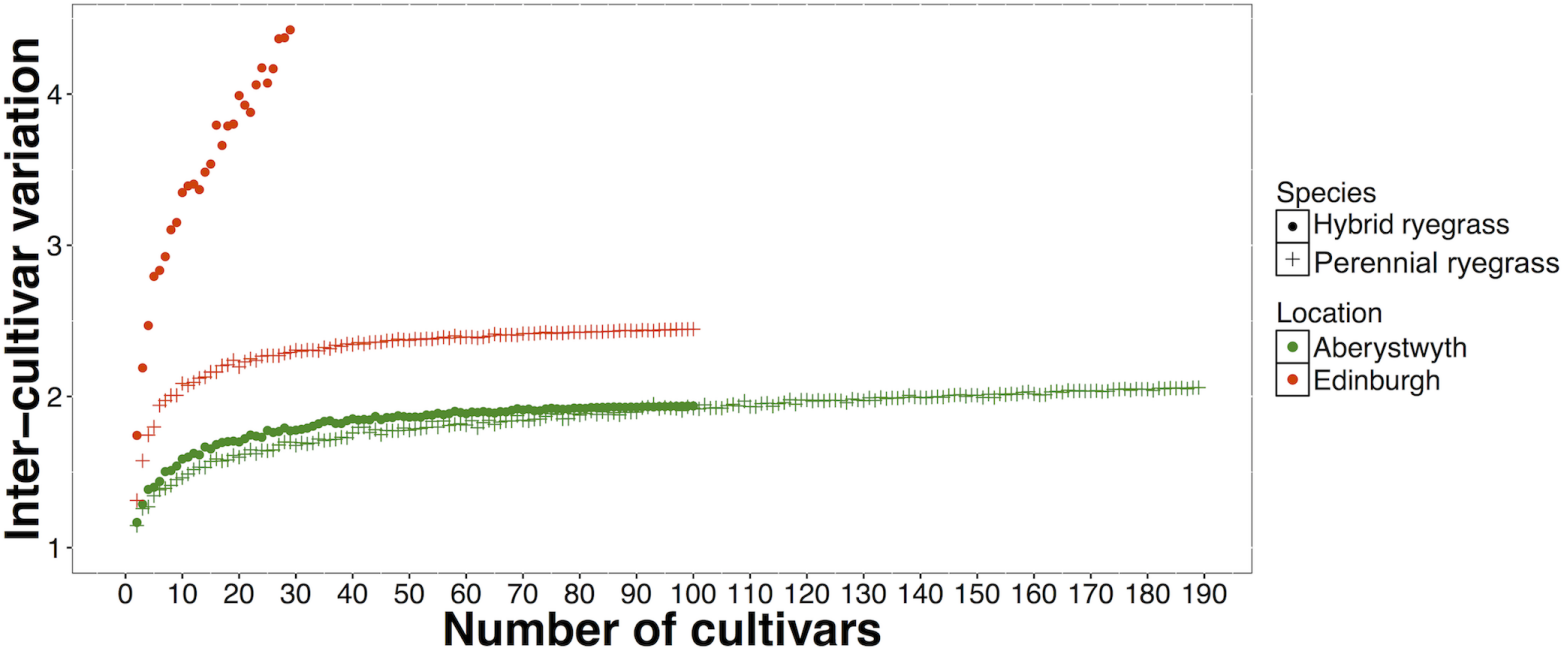
Inter-cultivar variation in strontium concentration ratios for 2 to maximum number of cultivars. Mean value of 100 simulations is shown.

It is likely that inter-cultivar variation will increase with increasing numbers of cultivars in an experiment, until the full genetic range has been represented. To test this, inter-varietal variation was calculated for two randomly selected cultivars. This was repeated in increments of two (4 cultivars, 6 cultivars, 8 cultivars etc….) to the maximum population size (max = 189 cultivars). This process was iterated 100 times and the means of these iterations were calculated and plotted (Cs: Fig 5; Sr: Fig 6). Simulations were not performed for Italian ryegrass and tall fescue, as there were too few cultivars for the simulations to be meaningful. All the curves derived from these simulations, apart from hybrid ryegrasses grown in Edinburgh (Fig 6), appear to be approaching a plateau at which the inter-cultivar variation would reach a maximum, which suggests that we have sampled nearly enough cultivars to be close to the full range. However, it is not possible to estimate this plateau accurately as the curves are constrained by the number of cultivars included in the experiments.

## Discussion

### The rationale of the study

Despite the importance of the soil-plant-milk pathway to human radioactive dose following nuclear accidents (e.g. [31]), and the agricultural importance of fodder crops (e.g. temporary grasslands cover nearly 1.4 million ha in the UK; [32]), research into exploiting variation in radiocaesium and radiostrontium uptake by different plant cultivars has focussed mainly on human food crop species [12]. Of these studies, most included fewer than 20 cultivars [12], exceptions being Penrose *et al*., 2016 [33] (70 cultivars; *Brassica oleracea*) Ohmori *et al*., [34] (85; *Oryza sativa*) and Moiseev *et al*., [35] (99 cultivars; *Pisum sativum*). Therefore, to our knowledge, this study, where a total of 397 cultivars from four common forage grass species (hybrid ryegrass (*Lolium perenne* L. x *Lolium multiflorum* Lam.; 101 cultivars), perennial ryegrass (*Lolium perenne* L.; 269 cultivars), Italian ryegrass (*Lolium multiflorum* Lam.; 17 cultivars) and tall fescue (*Festuca arundinacea* Shreb.; 10 cultivars) is the largest study to investigate inter-cultivar variation in Cs and Sr uptake by plants.

### Cultivars with consistently low Cs and Sr concentration ratios

We found three hybrid ryegrass cultivars that had consistently low Cs concentration ratios between four harvests over two years at two sites. We also found two cultivars that had consistently low Sr concentration ratios and one cultivar that had consistently low Cs and Sr concentration ratios, despite Cs and Sr having very different uptake mechanisms.

Concentration ratios were found to be lower in spring cuts than in summer cuts, which was consistent with findings of Salt et al. [36], who found almost 10-fold difference in stable Cs concentrations in bent grass (*Agrostis capillaris*) between both immature and mature leaves cut in mid-July and end of August. Seasonal effects on Cs uptake have been attributed to temperature, rainfall or soil moisture [37], which of these (or a combination) is responsible for the seasonal variation observed in this study is not clear. More research is required to elucidate the effect of these in UK conditions.

### Inter-species variation in Cs and Sr

Our findings suggest that substitution of one forage grass species by another ‘safer’ species with a lower CR [10], could be a remediation strategy for contaminated areas. Tall fescue cultivars were found to have lower Cs CRs (0.04–0.07) and Sr CRs (0.88–1.15) than most hybrid ryegrass, perennial ryegrass and Italian ryegrass cultivars. We therefore, propose that tall fescue could be considered a good substitute for *Lolium* forage grasses following a contamination incident. However, tall fescue is known to have lower dry matter digestibility and voluntary intake in comparison to *Lolium* species, although percentage dry matter and crude protein have been found to be similar for tall fescue and perennial ryegrass and dry matter yield has been found to be higher than perennial ryegrass [38]. Therefore, the effect of using tall fescue instead of *Lolium* for forage on agronomic factors such as yield and quality parameters such as digestibility need to be evaluated prior to implementation of this as a remediation strategy.

### Optimal number of cultivars

Our results suggest that increasing numbers of cultivars generally occurs concurrently with an increase in inter-cultivar variation, which implies that as the number of cultivars in an experiment increases, the likelihood of finding a larger variation between cultivars also increases. The inter-cultivar variations found in these experiments (Cs = up to 14-fold; Sr = up to 4.4-fold) were generally higher than those found in Alexakhin [39] (^137^Cs inter-cultivar variation = 1.4–4.5 fold), where between 4–15 cultivars from 10 plant species were studied and Penrose *et al*. [12] (Cs inter-cultivar variation = 1.8; Sr inter-cultivar variation = 2.0) in which 115 experiments including between 2 and 28 cultivars (Cs) and 2 and 99 cultivars (Sr) from over 50 species were collated. Previous studies that have included a greater number of cultivars, for example Ohmori *et al*., (2014) [34], which investigated variation in ^137^Cs activity concentrations among 85 rice (*Oryza sativa*) cultivars and Penrose *et al*., (2016) [33], which investigated variation in ^137^Cs and ^90^Sr concentration ratios in *c*. 70 *Brassica oleracea* cultivars have found larger inter-cultivar variation (Ohmori *et al*., 2014 [34] = *c*.10-fold; Penrose *et al*., 2016 [33]; ^137^Cs = 35-fold, ^90^Sr = 23-fold).

In this study, the relationship between the number of cultivars and the inter-cultivar variation in Cs and Sr concentration ratios was different for each of the species-location combinations, suggesting that the relationship is unique to each species in each location. More research on linkages between crop genetics, soil characteristics and environmental or management conditions is needed for this to be further elucidated. Whilst this study included a large number (29–189) of hybrid ryegrass and perennial ryegrass cultivars, it is likely that we would have observed larger inter-cultivar variation in Cs and Sr concentration if more cultivars had been studied. It is therefore recommended that studies in the future regarding inter-cultivar variation in Cs and Sr include as many cultivars as possible.

### Inter-cultivar variation in Cs and Sr

Our results suggest that cultivar substitution could be an effective remediation strategy for contaminated grasslands, and could reduce transfer of Cs to forage by up to 14-fold, and Sr by up to approximately 4-fold. To our knowledge, only two other studies (Øhlenschlæger & Gissel-Nielsen, [14]; [15]) have considered variation in Cs uptake between forage grass cultivars. Both papers, in which six studies were carried out, included only two perennial ryegrass cultivars (Darbor and Partoro). The inter-cultivar variation in ^137^Cs uptake ranged from 1.0–1.3 in their studies (Øhlenschlæger & Gissel-Nielsen, [14]; [15]), which was lower than the inter-cultivar variation in Cs found in our study (up to 14-fold). It is likely that the greater variation between cultivars in our study is due to the greater number of cultivars included.

The maximum reduction in Cs and Sr (Cs = up to 14-fold; Sr = up to 4.4-fold) by planting ‘safer’ cultivars with lower CRs are similar to—if not greater than—the existing mechanical and chemical soil amendments currently used for remediation of contaminated agricultural land. Mechanical soil amendment techniques such as ploughing can reduce radiocaesium and radiostrontium transfer to plants by up to 4-fold, whilst soil amendments such as liming or the addition of organic matter can reduce transfer by 1.5–4.0 fold and 1.3–3.0 fold, respectively [7]. Application of mineral fertilisers can reduce transfer of radiocaesium by 1.3-16- fold [40] and radiostrontium by up to 2.0-fold [41] depending on the soil type. As noted above, for Cs there are additionally effective binders which can be administered to animals to reduce the uptake of radiocaesium from the gut and hence transfer to milk and/or meat [8], though there are no feasible binders for Sr. Whilst dietary calcium levels can be manipulated to reduce radiostrontium transfer to milk it is likely that a maximum reduction of radiostrontium of at least *c*. two-fold could be achieved in most farming systems without exceeding recommended dietary calcium intakes [40].

Cultivar-selection could be used in conjunction with other existing strategies to add additional reductions in Cs and Sr transfer to food products. Farmers are advised to reseed 10–15% of their grassland per year, with priority given to weed-affected fields and fields with reduced yield. Therefore, reseeding early following a nuclear incident would not be very different from normal practice. Furthermore, it is likely that following an incident low-Cs and low-Sr seeds would be subsidised, reducing the cost to the farmer of reseeding. Reseeding involves both ploughing and fertilisation, and therefore these three remediation strategies used together may well reduce Cs and Sr transfer to livestock further. It is also likely that cultivar, or species substitution of forage grasses will be more acceptable to stakeholders than, for instance, the use of feed additives.

## Conclusions

Using existing ongoing grass breeding experiments allowed us to undertake the largest and most comprehensive examination of inter-cultivar variation in Cs and Sr uptake in plants to date over two sites, sampled at multiple time points over two years.

All tall fescue cultivars had lower Cs and Sr CRs than the majority of cultivars from the other three species, and therefore could be a candidate species for species substitution following a contamination incident.

Three (from 17) hybrid ryegrass cultivars were found to have consistently low Cs concentration ratios, two cultivars were found to have consistently low Sr concentration ratios, one cultivar was found to have lower Cs and Sr concentration ratios over time and between sites. This is the first time, to the authors’ knowledge, that forage grass cultivars with consistently lower Cs and Sr concentration ratios have been identified. The identification of cultivars with consistently lower concentration ratios suggests that substitution of cultivars with high concentration ratios for cultivars with lower concentration ratios could be an effective remediation strategy in contaminated grasslands. However, the sites were relatively similar in terms of soil characteristics and climate, and therefore cultivars with low concentration ratios should be tested for consistency over sites with more diverse environmental conditions.

We have found that inter-cultivar variation increases with increasing numbers of cultivars included in the experiment. We suggest that future research calculating inter-cultivar variation in Cs and Sr endeavours to include the largest feasible number of cultivars in order to increase the chances of reaching the maximum inter-cultivar variation.

## Supporting information

S1 Fig Ratio of the mean concentration of 28 elements in unwashed samples to the mean concentration of 28 elements in washed samples. The black line indicates a 1:1 ratio, where there is no difference in concentration between the washed and unwashed samples.
(TIFF)

S1 Table Washing test and ICP-MS methods. (XLSX)

S2 Table Experiment number, plot area, species and variety number, sowing year, sowing and harvest date and K, Ca, Rb, Sr and Cs concentration (mg kg^-1^). (XLS)

S3 Table Minimum and maximum concentration ratios (CRs) and inter-varietal variation (IVV; maximum CR/minimum CR) for 17 hybrid ryegrass cultivars grown in Aberystwyth and Edinburgh. (XLSX)

S4 Table Rankings (*R*) of 17 hybrid cultivars grown in both Aberystwyth and Edinburgh. The lowest ranking (1) denotes the cultivar with the lowest concentration ratio, the highest ranking (17) refers to the cultivar with the highest concentration ratio.
(XLSX)

S5 Table Mean concentration of Cs, Sr, Ca, Mg and K (mg kg^-1^) in perennial ryegrass, Italian ryegrass, hybrid ryegrass and tall fescue. Results are shown for Aberstwyth and Edinburgh.
(XLSX)

S6 Table Mean concentration of Cs, Sr, Ca, Mg and K (mg kg^-1^) for perennial ryegrass, Italian ryegrass, hybrid ryegrass and tall fescue in each field. (XLSX)
